# An Ecological Model Using Promotores de Salud to Prevent Cardiovascular Disease on the US-Mexico Border: The HEART Project

**DOI:** 10.5888/pcd9.110100

**Published:** 2012-01-12

**Authors:** Hector Balcázar, Sherrie Wise, E. Lee Rosenthal, Cecilia Ochoa, Maria Duarte-Gardea, Jose Rodriguez, Diana Hastings, Leticia Flores, Lorraine Hernandez

**Affiliations:** University of Texas, School of Public Health, El Paso Regional Campus; University of Texas, School of Public Health, El Paso Regional Campus, El Paso, Texas; University of Texas at El Paso, Department of Public Health Sciences, El Paso, Texas; University of Texas at El Paso, Department of Public Health Sciences, El Paso, Texas; University of Texas at El Paso, Department of Public Health Sciences, El Paso, Texas; City of El Paso Department of Parks and Recreation, El Paso, Texas; YWCA El Paso Del Norte Region, El Paso, Texas; El Paso Community College, El Paso, Texas; Centro San Vicente Clinic, El Paso, Texas

## Abstract

**Background:**

To address cardiovascular disease risk factors among Hispanics, a community model of prevention requires a comprehensive approach to community engagement. The objectives of our intervention were to reduce cardiovascular disease risk factors in Hispanics living in 2 low-income areas of El Paso, Texas, and to engage the community in a physical activity and nutrition intervention.

**Methods:**

Drawing on lessons learned in phase 1 (years 2005-2008) of the HEART Project, we used an iterative, community-based process to develop an intervention based on an ecological framework. New community partners were introduced and community health workers delivered several elements of the intervention, including the curriculum entitled "Mi Corazón, Mi Comunidad" ("MiCMiC" [My Heart, My Community]). We received feedback from the project's Community Health Academy and Leadership Council throughout the development process and established a policy agenda that promotes integration of community health workers into the local and state workforce.

**Outcome:**

Collaboration with 2 new community partners, the YWCA and the Department of Parks and Recreation, were instrumental in the process of community-based participatory research. We enrolled 113 participants in the first cohort; 78% were female, and the mean age was 41 years. More than 50% reported having no health insurance coverage. Seventy-two (60%) participants attended 1 or more promotora-led Su Corazón, Su Vida sessions, and 74 (62%) participants attended 1 or more of the 15 exercise classes.

**Interpretation:**

HEART phase 2 includes a multilevel ecological model to address cardiovascular disease risk among Hispanics. Future similarly targeted initiatives can benefit from an ecological approach that also embraces the promotora model.

## Background

The increasing rates of cardiovascular disease (CVD) among Hispanics, particularly those living in US-Mexico border communities, are of great public health concern (1). Obesity, diabetes, and hypertension contribute to poor health in this region, where much of the population is Hispanic or of Mexican origin. Community health promotion and disease prevention models are needed to compensate for the large number of uninsured, underinsured, and disadvantaged people living in these communities.

Moving from a clinical model of care to a community model of prevention requires a comprehensive approach to community engagement (2-5). The National Institute for Minority Health and Health Disparities has invested in an 8-year initiative introducing community-based participatory research (CBPR) to engage academia and communities in setting up programs with an ecological approach to health promotion and disease prevention. HEART (Health Education Awareness Research Team) is an example of one such collaborative effort; this program uses community health workers (promotoras de salud) in Hispanic communities along the US-Mexico border.

HEART has completed a 3-year pilot test (phase 1) using promotoras in a community randomized trial with participants from 2 underserved areas of El Paso, Texas. The pilot test resulted in more awareness of CVD risk factors among Hispanics, greater confidence in the control of these factors, and improved dietary habits (4,6) among trial participants. HEART phase 2 (2009-2013) will serve the same communities served by phase 1 (3,4,7-14). The objectives of phase 2 are 1) to reduce CVD risk factors among Hispanics and 2) to engage the community in an environmental restructuring initiative that focuses on nutrition and exercise. The environmental restructuring is designed to promote community use of existing physical activity and nutrition facilities and to integrate promotores into public-sector settings such as public parks, to address cardiovascular health promotion and CVD prevention.

## Methods

### Ecological framework for HEART phase 2

The conceptual framework for HEART phase 2 incorporates an ecological approach, whereby the environment of communities is enhanced and restructured (14-17). Our decision to incorporate the ecological approach was guided by empirical evidence for using parks and recreation facilities to implement physical activity and nutrition programming (17,18). The National Heart, Lung, and Blood Institute's Hearts N' Parks Y2K program — which integrated nutrition and physical activities into the North Carolina parks and recreation departments — also supports this approach (19).

We identified change agents at 5 levels: individual, interpersonal, organizational, community, and policy (Figure). At the individual level, the change agents are HEART participants who engage in the MiCMiC family of programs. The interpersonal level is represented by the HEART promotoras, and the participant's family, friends, and social networks. At the organizational level, we engaged new partners: the YWCA, and the Parks and Recreation Department of the City of El Paso (Parks and Recreation). The Community Health Advisory Council (CHAC) developed during phase 1 represents the policy agenda.

**Figure.**
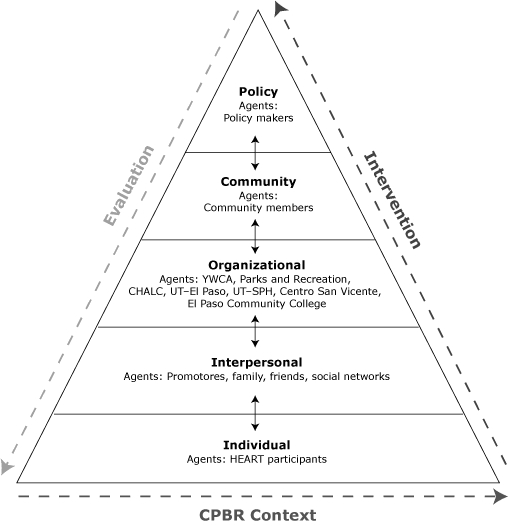
Phase 2 Conceptual Framework. Abbreviations: Parks and Recreation, Parks and Recreation Department of the City of El Paso; CHALC, Community Health Academy and Leadership Council; UT, University of Texas; SPH, School of Public Health; CPBR, community-based participatory research. Adapted from Bartholomew et al (15).

### Working with partners

The Community Health Advisory Council was renamed the Community Health Academy and Leadership Council  and was expanded to include members of the YWCA and the Parks and Recreation Department. It also included long-standing council members and Mexican American community members. We recruited new members through informal meetings. We established a Leadership Academy to support the Leadership Council and the promotoras hired to serve the intervention.

The University of Texas at El Paso initiated institutional agreements describing terms and responsibilities for each partner institution, which were signed by the designated officer at each institution. The research team held monthly meetings with the Leadership Council throughout the planning period and the intervention.

Parks and Recreation was instrumental in the coordination of park use, sound amplification, and other required city permits or reservations necessary for conducting MiCMiC activities outdoors and within recreation centers. We made arrangements with the Centro San Vicente clinic partner to use its teaching kitchen facility and with a popular grocery store chain to conduct heart-healthy grocery store tours.

### Promotoras training and preparation

We hired 3 certified promotoras through the YWCA. In addition to a high school diploma or equivalent, each promotora was required to possess or obtain Texas state community health worker certification within 30 days of employment. Additional requirements were a minimum of 2 years' experience in community project work, health-related service, cardiovascular health, or CBPR. Computer skills, ability to exercise, and training in CPR and first aid were also required.

Promotoras participated in a 2-week Basic Skills Leveling course developed by the El Paso Community College Community Health Worker/Promotores de Salud program for 45 credit hours. Learning modules focused on basic skills for reducing and preventing CVD in Hispanics, capacity-building strategies, tools for identifying community resources, and advocacy. Evaluation methods were pretests and posttests, assignments, student presentations, and an exit exam.

Additionally, a bilingual curriculum development specialist from HEART phase 2 trained the promotoras to conduct each activity of the MiCMiC curriculum, including heart-healthy cooking demonstrations. Parks and Recreation provided training and certification in proper food-handling techniques, and promotoras participated in in-house training on the YWCA regulations as full-time employees. All promotoras were also thoroughly trained in preintervention and postintervention data collection techniques.

### MiCMiC program planning

Assembly of the final set of MiCMiC programs was based on the approach proposed by Stokols (14,16) and using social cognitive theory (20) to highlight a multicomponent conceptual model emphasizing the influence of social ecology on individual behavior. We engaged the Community Health Academy and Leadership Council in a discussion of these constructs.

The curriculum development specialist conducted a cross-sectional inventory at the YWCA and Parks and Recreation locations in the community to assess human and physical resources and current use of services and community programs. The inventory included facility tours and interviews with administrators and staff, and site visits to 3 recreation centers, 1 senior center, 4 city parks, 1 YWCA branch location, and 6 elementary schools housing partner after-school programs. Patron and staff satisfaction with programming and perceived barriers to use were also assessed. We selected locations and programs for phase 2 on the basis of recommendations from YWCA and Parks and Recreation administrators, taking into consideration the following factors: availability (selection of locations receiving low to moderate use, staff willingness to participate), accessibility to participants (additional costs to participants, safety, location), and applicability to the intervention (heart health-related programming, relevance to targeted domains).

MiCMiC integrated several best practice methods identified by the CDC Task Force on Community Preventive Services, specifically the following: 1) providing self-management education in community meeting places for adults with type 2 diabetes (21), 2) creating highly visible community-wide campaigns that encourage physical activity (18), 3) providing access to existing local exercise facilities (18), 4) setting up walking groups to offer social support and fellowship (18), 5) using interventions individually tailored to participants' preferences and physical ability (18), and 6) improving participants' goal-setting and self-monitoring skills (18). These best practices matched recommendations from HEART phase 1 (4,6).

MiCMiC included the Su Corazón, Su Vida classroom-based curriculum from phase 1 (6), as well as new community partner activities selected by the curriculum development specialist, mainly instructor-led land and water aerobics classes at the YWCA. Each program type was categorized as focusing on nutrition or exercise. In addition, supplemental activities included 1) coffee talks (charlas), 2) heart-healthy cooking demonstrations, 3) heart-healthy grocery shopping tours, 4) Latin dance aerobics in the parks, 5) family soccer games, 6) family swim, and 7) walking groups in city parks that emphasize peer support and the "buddy system."

### Development of participant minimum expectations

**Table:** Box 1. Minimum Expectations for Participation

**Activity**	**Expectations per Month**	**Total per Intervention (4 months)**
**Lifestyle – Nutrition** Su Corazón, Su Vida	1 session	4 sessions

**Environment – Nutrition**	1 session	4 sessions
Coffee talks (charlas)		
Heart-healthy cooking demonstrations		
Heart-healthy shopping – grocery store tours		

**Lifestyle and Environment – Exercise^a^**	4 sessions	16 sessions
YWCA aerobics classes		
Family soccer		
Latin dance aerobics in the parks		
Walking groups in the parks		
Swimming in the parks		

**Free Choice (choose any activities from above)**	1 session	4 sessions
**TOTAL**	7 sessions	28 sessions

a Beginner, intermediate, and advanced levels of difficulty available.

**Table:** Box 2. Change Strategies and Evaluation Methods, by Ecological Level

**Level of HEART Intervention**	**Evaluation Method**
1*.* Individual: HEART participant	Pre- and postparticipant survey questionnaire to evaluate the impact of the MiCMiC 4-month intervention for the different study cohorts. Survey covers various heart-health domains including knowledge, beliefs, attitudes, perceptions and intentions, self-efficacy, social norms and outcomes such as blood pressure, BMI, waist circumference, dietary behaviors and 3-minute step test for heart rate.

2. Interpersonal: promotoras, family, friends, social networks	Pre- and postsurvey questionnaire as in level 1.
	Reflection notes and focus groups with the promotoras during pilot and cohort intervention.

3. Organizational: HEART Partners: Community Health Academy and Leadership Council, YWCA, Parks and Recreation Department, University of Texas-El Paso, University of Texas School of Public Health, Centro San Vicente, El Paso Community College	HEART partnership self-assessment instrument^a^ to assess effectiveness of Community Health Academy and Leadership Council meetings, outreach, trainings, and relationship with elected officials, as well as satisfaction with Community Health Academy and Leadership Council partnership development and strategic planning.
	Reflection notes and discussion session for YWCA and Department of Parks and Recreation.
	Community Health Academy and Leadership Council strategic planning and development of vision and mission statements.
	Community Health Academy and Leadership Council meetings' minutes discussion and self-reflection.

4. Community	Community telephone survey to test the impact of MiCMiC at the community level. Pre- and postintervention telephone survey to be conducted in intervention community and a control community. The survey evaluates perception and use of community health workers, perception of community resources for exercise and healthy living, dietary behavior, diabetes, hypertension, drinking, smoking, and basic demographics.
	HEART participant sign-up sheet available at final ceremony to identify leaders to continue heart-healthy activities in the community, post-intervention (ie, walking groups, aerobics activities, cooking clubs).

5. Policy: policy makers	Development of a policy agenda for HEART and documentation of actions carried out on that agenda.

a Adapted from the instrument developed by Butterfoss (22).

The HEART phase 2 research team developed a minimum expectation of what constitutes a 4-month intervention for participants once they are enrolled in MiCMiC (Box 1). An incentives schedule listed awards for completing the minimum expectations as well as intermediate milestones.

We developed a "passport" tool in English and Spanish that outlines the MiCMiC activities to be accomplished by participants during a 4-month period of intervention, in addition to the minimum expectations for the nutrition and physical activities. Participants record their activities in the passport.  The passport also includes a section for recording clinical measures. Promotoras encourage participants to bring their passports to the MiCMiC activities.

MiCMiC consists of 5 cohorts of 100 participants each. Each cohort participates in a 4-month intervention based on a pre-post design with 3 data collection points: baseline, 4 months, and 10 months (6 months post-intervention). Hispanic adults aged 18 years or older who resided in the 2 selected zip codes, were not planning to move from the area in the next 10 months, and were able to participate in the physical activities of MiCMiC were eligible. Recruitment was conducted by promotoras at community health fairs, the YWCA, recreation centers, Centro San Vicente, through personal contacts and referrals, and radio and TV Spanish programming.

HEART policy makers aim to integrate promotores into the local and state workforce. HEART's vision statement reflects this aim: *"An El Paso where both community health workers and members of the community work together to promote wellness and a heart-healthy environment."* HEART held several focus workshops with local employers and promotoras to identify key issues related to building the workforce. Among issues identified were building understanding of promotoras by employers, increasing unity among promotoras, and developing the promotora network. An ad hoc HEART-led group was established, including representatives of the HEART Community Health Academy and Leadership Council, promotora networks, and other area stakeholders who are working together to develop the promotora workforce. This group is taking the lead on hosting a community stakeholders' meeting to develop a 5-year strategic plan for promotora workforce development in the El Paso area.

We are using several methods to evaluate HEART phase 2 (Box 2).

## Outcome

In the spring of 2010, we conducted a 6-week pilot among 37 participants for HEART phase 2. The purpose of this pilot was to test the MiCMiC schedule of activities at partner locations, promotora readiness, and preparation for participant enrollment and data collection. Participants were given 4 months of free access to the YWCA where the Su Corazón, Su Vida classes and most of the exercise activities were delivered. Enrollment consisted of completing consent, the HEART participant questionnaire (administered by promotoras), clinical measures, a tour of the YWCA facility, and a kick-off meeting with the research team.

A total of 18 participants completed the HEART questionnaire at the conclusion of the pilot. Ten of those also participated in postpilot focus groups and exit surveys. Feedback was obtained regarding satisfaction with MiCMiC activities, schedule, and the usefulness of the HEART passport. Overall, we received positive reviews of the program; all the participants stated that they would recommend this program to a family member or friend.

The HEART Community Health Academy and Leadership Council was instrumental in providing community involvement and facilitating feedback in the planning and implementation of MiCMiC. Monthly Community Health Academy and Leadership Council meetings allow continued dialogue between the research team and other partners. Also, the research team developed a quarterly newsletter that reports progress to HEART participants.

Postintervention data collection is currently under way for the first cohort intervention. For cohort 1, a total of 113 participants from our target intervention area were enrolled and successfully completed consent, the participant survey questionnaire, and baseline clinical measures throughout May and June of 2010. At baseline, 78% of participants were female, and the mean age was 41 years (Table). More than 60% of participants reported Mexico as their birthplace, and 38% reported their birthplace as the United States. Mean length of US residence was 24 years, and most participants reported Spanish as their preferred language (92%). More than 50% reported having no health insurance coverage, and only 36% reported being employed. Mean years of education attained was 12, and more than 40% of participants reported an annual family income of less than $10,000, and another 30% reported an income of $10,000 to $20,000.

More than 20% reported never having had their cholesterol checked. Approximately 72% of participants reported they had had their blood pressure checked within the past year. Approximately 27% reported never having been screened for diabetes.

More than 60% of participants reported they do not exercise at least 30 minutes, 3 times per week (Table). More than 70% reported not consuming at least 5 fruits and vegetables per day. Most participants (88%) did not smoke.

Approximately 25% of participants reported they have received a positive diagnosis of hyperlipdemia by a health care provider at some time in the past; 19% have been told they have high blood pressure; and 13% had been diagnosed with diabetes.

MiCMiC activities were offered throughout the 4-month schedule. Seventy-two (60%) participants attended 1 or more (of 11 modules offered) promotora-led Su Corazón, Su Vida sessions, and 74 (62%) participants attended 1 or more of the 15 hour-long exercise classes offered at the YWCA partner location or used the YWCA gym or indoor pool for a workout. Physical activity events held at city park locations were attended by 59 participants at least once. These include Latin dance aerobics in the park, family sports, and walking groups. Supplemental nutrition activities such as the 5 charlas, heart-healthy grocery store tours, and 8 cooking demonstrations were attended by 35 (29%), 32 (26%), and 54 (45%) participants, respectively.

## Interpretation

The ecological model for prevention of CVD is the future of public health promotion. This ecological model must be culturally competent and must be a good fit for the community. HEART phase 2 embraces the promotora model as an important component of this ecological model. The model addresses each level, which is important, as others may concentrate only on one. Building such a comprehensive model is a challenge.

## Figures and Tables

**Table.** Baseline Demographic Characteristics of Participants in the HEART Project, El Paso, Texas, 2010

**Characteristic**	**Cohort 1, n (%), (n = 113)^a^**
**Female sex**	84 (78)
**Age, mean (SD), y**	41 (12)
**Birthplace**	
Mexico	70 (62)
United States	43 (38)
**Years of residence in United States, mean (SD)**	24 (15.5)
**Language preference**	
English	9 (8)
Spanish	104 (92)
**Years of educational attainment, mean (SD)**	12 (3.7)
**Employed**	40 (36)
**Financial status**	
Very well off	0
Well off	6 (5.5)
Getting by	71 (64.5)
Not getting by	33 (30)
**Annual family income, $**	
<10,000	46 (41.8)
10,000 to <20,000	33 (30)
≥20,000	31 (28.2)
**No health insurance**	62 (55)
**Marital status**	
Married/living with a partner	57 (50.9)
Widowed/separated/divorced	23 (20.5)
Never married	32 (28.6)
**No. of people in household, mean (SD)**	
Adults	2.25 (.7)
Children	2.02 (1.4)
**Ever diagnosed with hyperlipidemia**	28 (24.8)
**Ever diagnosed with hypertension**	23 (19.5)
**Ever diagnosed with diabetes**	15 (13.4)
**Screened for hyperlipidemia**	
Never	27 (23.9)
Within the past year	62 (54.9)
Within the past 2 years	12 (10.6)
3 or more years ago	12 (10.6)
**Had blood pressure checked**	
Never	18 (15.9)
Within the past year	81 (71.7)
Within the past 2 years	7 (6.2)
Within the past 3 years	7 (6.2)
**Screened for diabetes**	
Never	31 (27.4)
Within the past year	64 (56.6)
Within the past 2 years	6 (5.3)
3 or more years ago	12 (10.6)
**Exercising 30 min 3 times/wk**	42 (37.8)
**Eating 5 fruits and vegetables/d**	33 (29.7)
**Smoking**	
Never	73 (64.6)
Currently smoking	13 (11.9)
Within the past 30 days	14 (13)

Abbreviation: SD, standard deviation.

a All values are reported as n (%) unless otherwise indicated. Values for each variable may not correspond to the cohort total n because of missing responses. Percentages may not total 100 because of rounding.
